# Performance and Impact on Initial Antibiotic Choice of Direct Identification of Pathogens from Pediatric Blood Culture Bottles Using an In-House MALDI-TOF MS Protocol

**DOI:** 10.1128/spectrum.01905-21

**Published:** 2021-12-22

**Authors:** Andrés Pérez-López, Nazik Elamin, Rhanty Nabor, Sarah Dumindin, Diane Roscoe, Mohammad Rubayet Hasan, Mohammed Suleiman, Patrick Tang

**Affiliations:** a Division of Microbiology, Department of Pathology, Sidra Medicine, Doha, Qatar; b Weill Cornell Medical College in Qatar, Doha, Qatar; Children’s Hospital Los Angeles, University of Southern California

**Keywords:** MALDI-TOF, blood culture, bloodstream infections, children

## Abstract

The performance and early therapeutic impact of direct identification by matrix-assisted laser desorption ionization–time-of-flight mass spectrometry (MALDI-TOF; DIMT) on pediatric blood culture bottles using in-house-developed methods to obtain microbial pellets for spectrometric analysis have seldom been studied. During a 2-year period (June 2018 to May 2020), DIMT was performed on broths from positive pediatric blood culture bottles using an in-house-developed method. Organism identifications with a score of ≥1.6 were notified to treating clinicians. Therapeutic modifications that occurred after the communication of DIMT were reviewed through the electronic medical records. DIMT was performed on 530 pediatric positive blood culture bottles. Among 505 monomicrobial bottles, identifications from 298 (97.7%) deemed as bloodstream infections (BSI) and 189 (94.5%) as contaminations had DIMT notified to clinicians. All identifications were correct except for one Streptococcus mitis incorrectly reported as Streptococcus pneumoniae. Therapy modifications resulting from DIMT occurred in 27 (8.3%) patients with BSI. Deescalation from effective or ineffective broad-spectrum regimens occurred mainly in Enterococcus faecalis bacteremia, whereas appropriate escalation from an ineffective regimen with narrower spectrum occurred mainly in bacteremia caused by AmpC-β-lactamase-producing *Enterobacterales*. Escalation therapy was instituted significantly faster than deescalation therapy (median time, 0.75 versus 10.5 h [*P = *0.01]). DIMT also enabled clinicians to confirm contamination in nearly one-half of patients with contaminated blood cultures. Our DIMT method applied to positive pediatric blood culture bottles demonstrated reliable performance for the rapid identification of pathogens. Our DIMT approach allowed therapeutic optimization in BSI, especially involving microorganisms with intrinsic antibiotic resistance, and was helpful in the early identification of likely contaminants.

**IMPORTANCE** We demonstrate the performance and early impact on the antimicrobial management of bloodstream infections of an inexpensive, in-house preparation method for direct identification of bloodstream pathogens in pediatric blood culture bottles by matrix-assisted laser desorption/ionization–time-of-flight mass spectrometry.

## INTRODUCTION

Matrix-assisted laser desorption ionization–time-of-flight mass spectrometry (MALDI-TOF MS) has now become an indispensable part of routine, microbial identification procedures in the clinical microbiology laboratories. MALDI-TOF MS has accelerated the turnaround time of bacterial and fungal identification from days to minutes, allowing early initiation of appropriate antimicrobial therapy and infection control measures. Apart from identification of microorganisms from colonies, MALDI-TOF MS is widely used for direct identification of pathogens from positive blood culture bottles (1, 2). One method for direct identification by MALDI-TOF (DIMT) involves the Sepsityper kit (Bruker Daltonik, Bremen, Germany), a commercial assay designed to remove substances interfering with MS from the bottle, but it is costly, leading many laboratories to develop different in-house preparation methods (1).

The performance of direct identification by MALDI-TOF (DIMT) on positive blood cultures using both the Sepsityper kit and in-house pellet preparation methods has been extensively evaluated in adults and in children to a lesser extent (3–13). However, the early impact on antibiotic choices of direct identification of pathogens from positive pediatric blood culture bottles without susceptibility data has seldom been studied (7).

The purpose of this study was to evaluate the performance of DIMT on positive pediatric blood culture broths processed using a laboratory-developed method to generate a microbial pellet. Furthermore, we sought to determine the frequency with which children with positive blood cultures had therapeutic changes prompted by DIMT.

## RESULTS

### DIMT performance.

During the study period, DIMT analysis was performed on 530 positive pediatric blood culture bottles, including 505 monomicrobial and 25 polymicrobial samples. A total of 324 (61%) bacteremic episodes were deemed clinically significant. Of them, 42 (13%) were categorized as community-acquired (CA), 58 (18%) as health care-associated (HCA), and 224 (69%) as hospital-acquired (HA), respectively.

Among 505 monomicrobial bottles, a correct identification to genus and species was obtained in 473 (93.7%) and 316 (62.6%), respectively. Of the 148 monomicrobial bottles with Gram-negative organisms, a correct identification to genus was obtained in 144 (97.3%) and to species in 129 (87.2%). Of the 349 monomicrobial bottles with Gram-positive organisms, 324 (92.8%) were correctly identified to genus and 187 (53.6%) to species (Table 1 and Table S1). Correct identification to species was significantly higher among Gram-negative organisms (*P < *0.001). Five viridans group streptococci (VGS) were misidentified. Two Streptococcus oralis bottles were erroneously identified as Streptococcus mitis and Streptococcus gordonii. One S. mitis bottle was misidentified as S. oralis, whereas 2 S. mitis bottles were misidentified as Streptococcus pneumoniae. Among monomicrobial bottles, 305 (60.4%) corresponded to clinically significant bacteremic episodes. Of these, 293 bottles (96.1%) and 216 bottles (70.8%) were correctly identified to the genus and species. Of the 200 monomicrobial bottles eventually deemed as contaminations, 178 (89%) and 100 (50%) were correctly identified to the genus and species. Rates of correct identification among monomicrobial bottles were significantly higher for those containing clinically significant isolates for both genus (*P = *0.002) and species (*P < *0.001).

**TABLE 1:** Results according to species of direct identification using an in-house MALDI-TOF MS protocol performed on 505 monomicrobial positive pediatric blood culture bottles^*a*^

Colony identification	*n*	Direct identification by MALDI-TOF scores			
		Correct ID score of >2, *n* (%)	Correct ID score of 1.7–1.99, *n* (%)	Score of <1.7, *n* (%)	False ID, *n* (%)
*Enterobacterales*	113	104 (92)	9 (8)	0	0
Nonfermenting Gram-negative rods	27	23 (85)	3 (11)	1 (4)	0
Fastidious Gram-negative coccobacilli	8	2 (25)	3 (38)	3 (38)	0
Staphylococci	256	130 (51)	116 (45)	10 (4)	0
Streptococci	61	44 (72)	15 (25)	2 (3)	5 (8)
Gram-positive rods	19	6 (32)	6 (32)	7 (37)	0
Other Gram-positive bacteria	13	11 (85)	1 (8)	1 (8)	0
Yeast	8	0	5 (63)	3 (38)	0

a ID, identification; false ID, discrepancy between DIMT and colony identification confirmed by 16S rRNA gene sequencing.

Altogether, 510 (96.2%) identifications were reported to clinicians. Of them, 509 (99.8%) were correctly reported taking into account that VGS and coagulase-negative staphylococci (CoNS) were reported only to the group level. Among patients with clinically significant monomicrobial bacteremic episodes, 298 (97.7%) had an identification reported to clinicians. All bloodstream infections (BSI) caused by Gram-negative organisms (142 isolates) and yeast (6 isolates) had a correct identification communicated. All except one BSI caused by Gram-positive organisms had a correct identification reported to clinicians (149/150; 99.3%). One Streptococcus mitis bottle was erroneously reported as Streptococcus pneumoniae. This blood culture bottle was collected from a patient with acute lymphoblastic leukemia, mucositis, and neutropenic sepsis. Two months later, another S. mitis bottle recovered from the same patient with neutropenic fever was misidentified as S. pneumoniae. However, since the Gram stain revealed Gram-positive cocci in long chains and the clinical picture was not consistent with pneumococcal bacteremia, DIMT was not reported. Among patients with monomicrobial nonsignificant bacteremic episodes, 189 (94.5%) identifications were reported to clinicians. All blood cultures considered to be contaminated had a correct identification communicated.

Among 25 polymicrobial bottles, a single pathogen was identified to genus in 22 bottles (88%) and to species in 18 bottles (72%). Twenty-three patients (92%) with polymicrobial bacteremic episodes had at least a predominant pathogen with a score value of ≥1.6 reported to clinicians, including 18 (94.7%) out of 19 clinically significant episodes.

DIMT had to be repeated on 54 bottles after room temperature incubation, including 30 (55.6%) eventually considered to be contamination. Forty-one repeated bottles (75.9%) eventually achieved an identification to the genus level, whereas 22 (40.7%) were identified to species level. A score of ≥1.6 was achieved in 44 (81.5%) repeated bottles, including 21 (47.7%) from BSI.

Nineteen blood culture bottles had a score value of <1.6, including 11 (57.9%) contaminated bottles (Table S2).

### Clinical impact.

**FIG 1:**
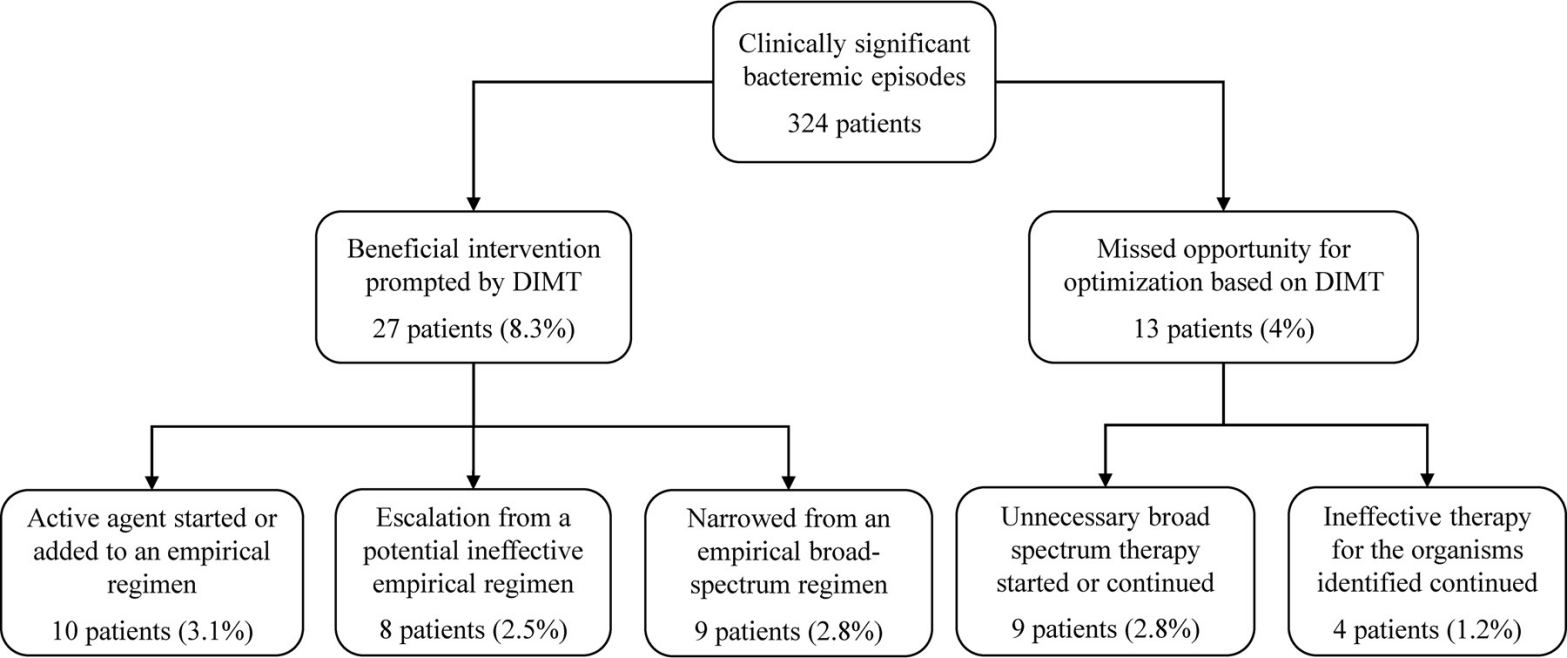
Clinical impact of direct identification by MALDI-TOF on clinically significant bacteremic episodes.

Twenty-seven (8.3%) patients with BSI had treatment modifications prompted by DIMT (Fig. 1 and Table S4). Therapy changes were led by the identification of Enterococcus faecalis (8 patients [29.6%]), AmpC-β-lactamase-producing *Enterobacterales* (7 patients [25.9%]), Pseudomonas aeruginosa and beta-hemolytic streptococci (3 patients [11.1% each]), Enterococcus faecium, Staphylococcus capitis, Staphylococcus aureus, Streptococcus pneumoniae, Klebsiella pneumoniae, and nontypeable Haemophilus influenzae (1 patient [3.7% each]). Nine patients initially started on effective or ineffective broad-spectrum empirical regimens were deescalated to targeted agents with a narrower spectrum, including 4 patients with Enterococcus faecalis narrowed from glycopeptides to ampicillin and 2 patients with the same species switched from ceftriaxone to ampicillin. Median time to deescalation was 10.5 h (interquartile range [IQR], 4 to 17.4 h). Eight patients were appropriately escalated from an ineffective empirical therapy to targeted agents with broader spectrum, including 4 patients with AmpC-β-lactamase-producing *Enterobacterales* escalated from ceftriaxone or piperacillin-tazobactam to meropenem. Median time to escalation was 0.75 h (IQR, 0.17 to 3.8 h). Institution of escalation therapy occurred significantly faster than narrowing (*P = *0.01). In addition, 5 (1.5%) previously untreated patients were started on an active agent for the microorganisms identified.

We also identified 13 additional clinically significant bacteremic episodes in which DIMT could have led to an optimization of empirical antimicrobial therapy (Fig. 1 and Table S5). All these patients were narrowed or switched only after susceptibilities were confirmed on colonies, including 5 patients with E. faecalis deescalated from glycopeptides to ampicillin, 4 patients with Candida albicans deescalated from amphotericin B or an echinocandin to fluconazole, 3 patients with AmpC-β-lactamase-producing *Enterobacterales* switched to meropenem, and 1 patient with group A Streptococcus switched from clindamycin to penicillin.

**FIG 2:**
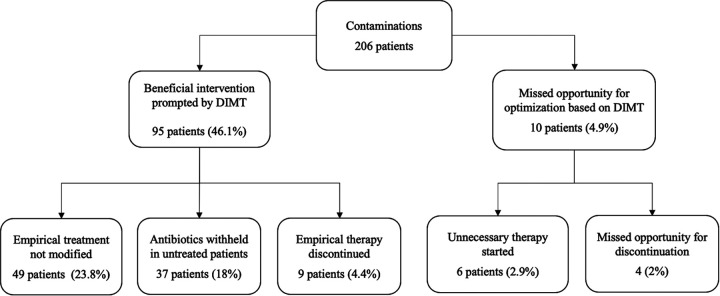
Clinical impact of direct identification by MALDI-TOF on contaminated blood cultures.

Overall, 95 (46.1%) of 206 bottles were deemed potentially contaminated based on DIMT, including 78 (38%) with CoNS (Fig. 2). Nine patients with bottles considered to be contaminated based on DIMT had an empirical therapy discontinued. Median time for discontinuation was 3.5 h (IQR, 0.5 to 12.5 h). In the pediatric emergency department (PED), 62 (60.2%) positive blood culture bottles eventually considered nonsignificant were already interpreted as contaminations based on DIMT. Among these patients, 30 (29.1%) empirically started on antibiotics did not have their therapy modified; 29 (28.1%) untreated patients continued without antibiotics and 3 (2.9%) had unnecessary treatment discontinued. CoNS was identified in 84% of bottles considered to be contaminated based on DIMT in the PED. Of the 62 contaminated bottles from the neonatal intensive care unit (NICU) and pediatric intensive care unit (PICU), 16 (25.8%) were interpreted as contaminations based on DIMT. Nine (15%) patients continued with the same regimen, two (3%) untreated patients had treatment withheld, and five (8%) patients had unnecessary treatment stopped. Contaminated bottles were significantly more likely to be interpreted as contamination based on DIMT by ED clinicians than by critical care clinicians (*P < *0.001).

## DISCUSSION

In the present study, the performance of DIMT on positive pediatric blood culture bottles using an in-house-developed method to obtain a microbial pellet for mass spectrometric analysis did not differ significantly from that of previous pediatric studies using the Sepsityper kit and in-house methods (7–11). In addition, our method was inexpensive, user-friendly, and easily integrated into a standard laboratory workflow. Of note, using our pellet preparation method, a successful identification was achieved in almost 90% of bottles processed immediately after removal from the continuous monitoring blood culture instrument. It was also noteworthy that more than 80% of the unsuccessful DIMT performed after the bottles were removed from the blood culture instrument had an organism identified when the assay was repeated after a short incubation period at room temperature, suggesting a persistent increase of bacterial concentration in an enriched broth that enhances the identification yield (12).

Likewise, the microbiology reporting approach adopted in our study yielded reliable identifications in the vast majority of patients, including those with positive blood cultures by Gram-positive cocci and yeast, two well-known limitations of mass spectrometry technology (1–3, 13–15). The only incorrect identification reported to clinicians was an S. mitis sample misidentified as S. pneumoniae. Although S. pneumoniae is reported only by the clinical microbiologist when the Gram stain morphology and clinical scenario are consistent with pneumococcal bacteremia, given the limitation of MALDI-TOF MS to differentiate species within the S. mitis group (3, 14, 15), this particular sample became positive during a night shift and was reported by a technologist prior to consultation with the microbiologist.

In our setting, DIMT allowed early optimization of empirical antibiotic therapy in children with BSI caused by a subset of species with predictable susceptibility patterns, such as E. faecalis, AmpC-producing *Enterobacterales*, beta-hemolytic streptococci, and Pseudomonas aeruginosa. In contrast, the impact of DIMT to reduce the empirical use of broad-spectrum antibiotics, namely, glycopeptides and carbapenems, was hampered by the high local prevalence of methicillin resistance in Staphylococcus aureus and extended-spectrum beta-lactamase (ESBL)-producing *Enterobacterales*. In fact, 28% of Escherichia coli and K. pneumoniae isolates recovered from blood cultures in this study were ESBL producers, including 5 isolates coproducing carbapenemases, whereas 32% of S. aureus isolates were resistant to methicillin (data not shown). Since the majority of BSI recorded were HCA or HA, our data suggest that DIMT without rapid molecular antimicrobial susceptibility testing can offer limited real-time opportunities to reduce the use of broad-spectrum antibiotics in children at greater risk for developing BSI from multidrug-resistant bacteria (16–18). Notably, the regimens for all BSI caused by C. albicans and almost 40% of E. faecalis BSI were narrowed once antimicrobial susceptibility testing results were available. Patients with candidemia remained on broad-spectrum antifungals after consultation with the pediatric infectious diseases (PID) physician because they were clinically unstable (19), whereas patients with enterococcal bacteremia were treated with glycopeptides following identification because the clinicians were unwilling to use a narrow spectrum agent based on results from a recently implemented in-house-developed assay (5, 6). It is interesting to note that therapy escalation prompted by rapid identification of bloodstream pathogens occurred significantly faster than deescalation. Although it is beyond the scope of our study to determine treatment decision-making attitudes and practices behind this difference, it could be speculated that switching to a broader effective agent from an ineffective empirical regimen with a narrower spectrum might have been considered a priority by attending physicians (17). Conversely, switching from a broad-spectrum regimen, particularly when the pathogen identified is appropriately covered, to a targeted agent with a narrower spectrum might have required further input from other clinical team members.

Our DIMT approach proved to be reliable also in the early identification of possible contaminations. Particularly when patients were seen in the PED, DIMT assisted clinicians when “Gram-positive cocci in clusters” were reported to resolve the dilemma of differentiating between S. aureus and CoNS (7). It was noteworthy that further telephone input after notification of identification was sought by emergency department (ED) clinicians in only 15% of contaminated blood cultures (data not shown). Conversely, DIMT was less helpful to rule out contamination for patients in our critical care units. Since the collection of paired blood cultures from patients with central lines in place is not a standard practice in NICU and PICU, this finding probably reflects the difficulty of interpreting the significance of a single positive blood culture with CoNS in the presence of central venous catheters (20).

Our study has several limitations. We could not assess the performance of our method on obligate anaerobic bacteria, since we did not register any bacteremic episode by these microorganisms during the study period. However, this finding was not surprising given that BSI associated with obligate anaerobes are uncommon in children (15). Another limitation was that our diagnostic approach and antimicrobial stewardship (AMS) interventions were not subjected to independent appraisal. Finally, the reduction in time to optimal antimicrobial therapy once direct identification was notified was not compared with a preinterventional group using a conventional blood culture processing method (15, 21).

In summary, DIMT using a laboratory-developed method to generate microbial pellets from positive pediatric blood culture bottles in combination with a modified cutoff value for notification provided rapid and reliable identification of pathogens. Although the lack of susceptibility data is currently a limitation of mass spectrometry, we believe that our DIMT approach combined with appropriate AMS interventions could help streamline antimicrobial therapy in children with BSI caused not only by pathogens with intrinsic resistance to antibiotics but also by other microorganisms in settings with low antibiotic resistance rates. Finally, our approach proved to be a helpful tool to reduce unnecessary antibiotic therapy for patients with contaminated blood cultures.

## MATERIALS AND METHODS

### Setting.

This prospective study was conducted over a 2-year period, between June 2018 and May 2020, at Sidra Medicine in Doha, a 400-bed tertiary women’s and children’s hospital with approximately 2,000 pediatric admissions and 75,000 visits to the PED per year. The hospital serves as a referral center for the pediatric population of the whole country for all medical subspecialties, including oncology, NICU, PICU, and surgical specialties, including cardiac surgery and neurosurgery.

### In-house MALDI-TOF protocol and implementation.

**FIG 3:**
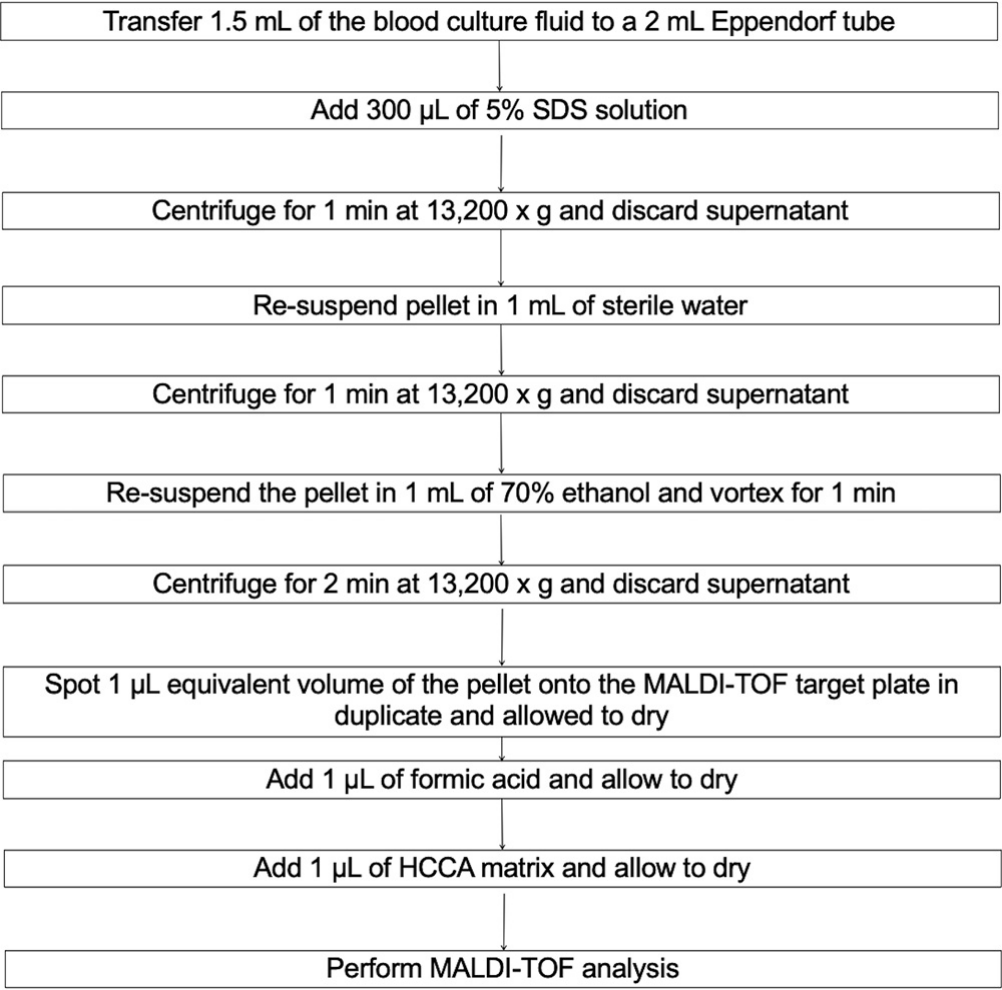
In-house pellet preparation procedure to perform direct identification of pathogens on pediatric blood culture bottles by MALDI-TOF MS.

DIMT analysis was performed on positive BD Bactec Peds Plus/F bottles (Becton, Dickinson, Franklin Lakes, NJ) from children aged <18 years. For each episode of bacteremia, one positive blood culture bottle would undergo DIMT analysis. Our stepwise sample preparation protocol based on blood cell lysis with sodium dodecyl sulfate and double protein extraction with ethanol and formic acid is depicted in Fig. 3. The spectrometric analysis was eventually performed using the MALDI Biotyper system (Bruker Daltonik, Bremen, Germany) in standard mode and analyzed with MALDI Biotyper Compass Explorer 4.1 software matched against BDAL library (Bruker Daltonik, Bremen, Germany). On average, the procedure required 30 min and the price per spot tested was £1.2.

Identification thresholds to genus and species levels were initially set at ≥1.7 and ≥2 according to the Bruker system. Nevertheless, any pathogen but VGS and CoNS with a score of ≥1.6 was considered successfully identified to the species level and consequently reported to the treating clinician. The taxonomic identification with the highest score was reported to clinicians. Direct identification of VGS and CoNS were reported only to the group level irrespective of the score. Subcultured colonies isolated on solid media were identified using MALDI-TOF and BD Phoenix panels (Becton, Dickinson, Franklin Lakes, NJ). Taxonomic discrepancies between DIMT and subcultured colonies from agar plates were resolved by 16S rRNA gene sequencing.

The DIMT procedure was performed simultaneously with Gram staining of all positive pediatric blood culture bottles, 24 h a day. DIMT was performed by the technologist responsible for processing blood cultures. If DIMT was unsuccessful, one additional attempt was made by a different technologist after an incubation period at room temperature ranging from 2 to 8 h. In addition to the Gram stain results, the DIMT results were also immediately phoned to clinicians, and the written report was accompanied by the comment “Preliminary identification based on a laboratory-developed method.” A senior attending clinical microbiologist reviewed all potentially clinically significant results between 7 a.m. to 11 p.m. and, when relevant, engaged the clinicians to review the clinical significance and antibiotic choice. Furthermore, telephone advice 24 h per day was available through the on-call PID consultant and clinical microbiology consultant. In this study, a CoNS isolated from a single blood culture bottle was considered significant if recovered from a child with central venous catheter in place and clinical symptoms, signs, and/or laboratory markers consistent with infection. VGS, *Corynebacterium* spp., or *Bacillus* spp. were considered contaminants unless isolated from two or more blood culture bottles in the appropriate clinical context.

### Clinical impact assessment.

Clinically significant bacteremic episodes were classified as CA, HCA, and HA infections as described elsewhere (22). The significance and classification of every positive blood culture in our institution are established during daily multidisciplinary meetings carried out by AMS, PID, infection prevention and control, and clinical microbiology team members.

To assess the real-time impact of our method on antibiotic decision-making in BSI, treatment modifications that occurred between the time of reporting DIMT and the identification of colonies on solid media were tracked in our electronic medical record system. Time to therapy change was calculated from the time DIMT was phoned to the attending clinician to the time therapy modification was electronically registered by the pharmacy. Only treatment modifications that required additional information beyond the Gram stain were recorded. Empirical therapy was considered effective if the microorganism identified was eventually susceptible *in vitro* to at least one antibiotic administered at the time DIMT was reported. Table S3 depicts potential beneficial treatment modifications identified in patients with clinically significant bacteremic episodes. Optimal treatment adjustments that occurred based on DIMT were divided into three categories: first, patients narrowed from an effective or ineffective broad-spectrum empirical therapy, second, patients that were appropriately escalated from an ineffective empirical therapy with narrower spectrum, and third, initiation of appropriate therapy in patients who were not treated with antibiotics at the time of reporting DIMT, or addition of an active agent to the initial ineffective empirical regimen. In addition, we recorded opportunities provided by DIMT that were missed by treating clinicians based on criteria presented in Table S3.

The impact of DIMT on contaminated blood cultures was assessed by dividing patients into 3 categories: first, patients who did not have their empirical antibiotic treatment modified, second, untreated patients in whom antibiotic treatment was withheld, and third, patients that had their empirical therapy discontinued.

### Statistical analysis.

The association between variables was assessed by the chi-square test with Fisher’s exact test when an expected value was less than 5. The time to therapy change was expressed as median with 25th percentile and 75th percentile IQR. Time differences between escalation and deescalation were compared using the Mann-Whitney U test. For all statistical analyses, a *P* value of ≤0.05 was considered significant.
