# Supplementary material for: Performance and Impact on Initial Antibiotic Choice of Direct Identification of Pathogens from Pediatric Blood Culture Bottles Using an In-House MALDI-TOF MS Protocol
Source: Microbiol Spectr. 2021 Dec 22;9(3):e01905-21. doi: 10.1128/spectrum.01905-21 (PMC8694180; doi:10.1128/spectrum.01905-21)
Supplement: SUPPLEMENTAL FILE 1 — Supplemental material. Download SPECTRUM01905-21_Supp_1_seq6.pdf, PDF file, 0.2 MB [file spectrum01905-21_supp_1_seq6.pdf]

Supplementary Table 1. Results according to species of direct identification using an in-house MALDI-TOF MS protocol performed on 505 monomicrobial positive pediatric blood culture bottles

		**Direct identification by MALDI-TOF**			
**Colony identification**	**n**	**Correct ID Score >2 n (%)**	**Correct ID Score 1.7-1.99 n (%)**	**Score <1.7 n (%)**	**False ID n (%)**
**Enterobacterales**	**113**	**104 (92)**	**9 (8)**	**0**	**0**
Klebsiella pneumoniae	**49**	**46 (94)**	**3 (6)**	**0**	**0**
Eschericia coli	**30**	**30 (100)**	**0**	**0**	**0**
***Salmonella* spp.**	**10**	**9 (90)**	**1 (150)**	**0**	**0**
Enterobacter cloacae	**9**	**7 (78)**	**2 (22)**	**0**	**0**
Serratia marcescens	**5**	**4 (80)**	**1 (20)**	**0**	**0**
Klebsiella oxytoca	**4**	**3 (75)**	**1 (25)**	**0**	**0**
Enterobacter aerogenes	**2**	**2 (100)**	**0**	**0**	**0**
Proteus mirabilis	**1**	**1 (100)**	**0**	**0**	**0**
Pantoea eucrina	**1**	**0**	**1 (100)**	**0**	**0**
Raoultella ornithinolytica	**1**	**1 (100)**	**0**	**0**	**0**
Klebsiella aerogenes	**1**	**1 (100)**	**0**	**0**	**0**

Colony identification	n	Correct ID Score >2 n (%)	Correct ID Score 1.7-1.99 n (%)	Score <1.7 n (%)	False ID n (%)
**Non-fermenting gram-negative rods**	**27**	**23 (85)**	**3 (11)**	**1 94)**	**0**
Pseudomonas aeruginosa	**19**	**18 (95)**	**1 (5)**	**0**	**0**
Ochrobactrum intermedium	**2**	**2 (100)**	**0**	**0**	**0**
Acinetobacter baumannii	**1**	**1 (100)**	**0**	**0**	**0**
Achromobacter xylosoxidans	**1**	**1 (100)**	**0**	**0**	**0**
Chryseobacterium indologenes	**1**	**1 (100)**	**0**	**0**	**0**
Elizabethkingia meningoseptica	**1**	**0**	**1 (100)**	**0**	**0**
Sphingomonas paucimobilis	**1**	**0**	**0**	**1 (100)**	**0**
***Stenotrophomonas maltophili*a**	**1**	**2 (100)**	**0**	**0**	**0**

Colony identification	n	Correct ID Score >2 n (%)	Correct ID Score 1.7-1.99 n (%)	Score <1.7 n (%)	False ID n (%)
**Fastidious gram-negative coccobacilli**	**8**	**2 (25)**	**3 (38)**	**3 (38)**	**0**
Haemophilus influenzae	**3**	**0**	**2 (67)**	**1 (33)**	**0**
Moraxella catarrhalis	**2**	**2 (100)**	**0**	**0**	**0**
Moraxella osloensis	**2**	**0**	**1 (50)**	**1 (50)**	**0**
Campylobacter coli	**1**	**0**	**0**	**1 (100)**	**0**

Colony identification	n	Correct ID Score >2 n (%)	Correct ID Score 1.7-1.99 n (%)	Score <1.7 n (%)	False ID n (%)
Staphylococci	256	130 (51)	116 (45)	10 (4)	0
Stapylococcus epidermidis	124	30 (24)	84 (68)	10 (8)	0
Staphylococcus hominis	52	42 (81)	10 (19)	0	0
Staphylococcus aureus	41	34 (83)	7 (17)	0	0
Staphylococcus capitis	21	13 (62)	8 (38)	0	0
Staphylococcus haemolyticus	12	8 (67)	4 (33)	0	0
Staphylococcus warneri	2	1 (50)	1 (50)	0	0
Staphylococcus saprophyticus	1	0	1 (100)	0	0
Staphylococcus caprae	1	0	1 (100)	0	0
Staphylococcus pettenkofei	1	1 (100)	0	0	0
Staphylococcus spp.	1	1 (100)	0	0	0

Colony identification	n	Correct ID Score >2 n (%)	Correct ID Score 1.7-1.99 n (%)	Score <1.7 n (%)	False ID n (%)
**Streptococci**	**61**	**44 (72)**	**15 (25)**	**2 (3)**	**5 (8)**
**Viridans group streptococci**	**24**	**16 (67)**	**8 (33)**	**1 (9)**	**5 (21)**
Enterococcus faecalis	**13**	**12 (92)**	**1 (8)**	**0**	**0**
Streptococcus pneumoniae	**11**	**6 (55)**	**4 (36)**	**1 (9)**	**0**
**Group A *Streptococcus***	**4**	**3 (75)**	**1 (25)**	**0**	**0**
**Group B *Streptococcus***	**4**	**3 (75)**	**1 (25)**	**0**	**0**
Abiotrophia defectiva	**2**	**1 (50)**	**0**	**1 (50)**	**0**
Enterococcus faecium	**1**	**1 (100)**	**0**	**0**	**0**
Streptococcus gallolyticus	**1**	**1 (100)**	**0**	**0**	**0**
Granulicatella adiacens	**1**	**1 (100)**	**0**	**0**	**0**

Colony identification	n	Correct ID Score >2 n (%)	Correct ID Score 1.7-1.99 n (%)	Score <1.7 n (%)	False ID n (%)
**Gram-positive rods**	**19**	**6 (32)**	**6 (32)**	**7 (37)**	**0**
***Bacillus* spp.**	**8**	**5 (63)**	**3 (38)**	**0**	**0**
***Corynebacterium* spp.**	**6**	**1 (17)**	**1 (17)**	**4 (67)**	**0**
***Actinomyces* spp**	**2**	**0**	**0**	**2 (100)**	**0**
Rothia dentocariosa	**1**	**0**	**0**	**1 (100)**	**0**
Leuconostoc mesenteroides	**1**	**0**	**1 (100)**	**0**	**0**
Microbacterium lacticum	**1**	**0**	**1 (100)**	**0**	**0**

**Colony identification**	**n**	**Correct ID Score >2 n (%)**	**Correct ID Score 1.7-1.99 n (%)**	**Score <1.7 n (%)**	**False ID n (%)**
**Other gram-positive bateria**	**13**	**11 (85)**	**1 (8)**	**1 (8)**	**0**
Micrococcus luteus	**12**	**10 (83)**	**1 (8)**	**1 (8)**	**0**
Kokuria kristinae	**1**	**1 (100)**	**0**	**0**	**0**

Colony identification	n	Correct ID Score >2 n (%)	Correct ID Score 1.7-1.99 n (%)	Score <1.7 n (%)	False ID n (%)
Candida spp.	8	0	5 (63)	3 (38)	0
Candida albicans	5	0	3 (60)	2 (40)	0
Candida metapsilosis	1	0	1 (100)	0	0
Candida parapsilosis	1	0	0	1 (100)	0
Candida tropicalis	1	0	1 (100)	0	0

Supplementary Table 2. Unsuccessful direct identification using an in-house MALDI-TOF MS protocol

**Colony ID**	**DIMT**	**DIMT score**	**Clinical significance**
Abiotrophia defectiva	NOIDP	NPD	Contamination
Candida tropicalis *Staphylococcus hominis*	*Candida tropicalis*	1.38	Significant
Corynebacterium afermentans	*Corynebacterium afermentans*	1.47	Contamination
Staphylococcus epidermidis	*Staphylococcus epidermidis*	1.54	Contamination
Staphylococcus epidermidis	*Staphylococcus epidermidis*	1.57	Contamination
Sphingomonas paucimobilis	*Sphingobium chlorophenolicum*	1.35	Contamination
Campylobacter coli	NOIDP	NPD	Significant
Corynebacterium spp.	NOIDP	NPD	Contamination
Staphylococcus epidermidis	*Staphylococcus epidermidis*	1.59	Significant

Brevundimonas diminuta *Acidovorax temperans*	*Gordonia rubripertincta*	1.26	Contamination
Staphylococcus epidermidis	*Staphylococcus epidermidis*	1.53	Significant
Candida parasilopsis	*Candida parasilopsis*	1.48	Significant
Haemophilus influenzae	NOIDP	NPD	Significant
Candida albicans	*Candida albicans*	1.53	Significant
Actinomyces odontolyticus	NOIDP	NPD	Contamination
Streptococcus pneumoniae	NOIDP	NPD	Significant
Rothia dentocariosa	NOIDP	NPD	Contamination
Moraxella osloensis	*Clostridium cochlearium*	1.34	Contamination
Corynebacterium spp	*Corynebacterium afermentans*	1.46	Contamination

Supplementary Tabe 3. Optimal de-escalating and escalating interventions identified in clinically significant bacteremic episodes based on DIMT

**De-escalation**	Glycopeptide or 3G/4G-cephalosporins to ampicillin for *Enterococcus faecalis*
	Piperacillin-tazobactam to ceftriaxone for *Haemophilus influenzae*
	Glycopeptide or 3G-cephalosporins to penicillin for beta-hemolytic streptococci
	Amphotericin B or echinocandin to fluconazole for *Candida albicans*
**Escalation**	3G-cephalosporin to glycopeptides for *Enterococcus faecium*
	Cloxacillin to glycopeptides for coagulase-negative staphylococci
	Ampicillin + gentamicin to piperacillin-tazobactam for *Pseudomonas aeruginosa*
	3G-cephalosporins or piperacillin-tazobactam to meropenem for AmpC beta-lactamase producers
	Ampicillin + gentamicin to meropenem for *Klebesiella pneumoniae*

Supplementary Tabe 4. Treatment optimization in blood stream infections prompted by direct identification by MALDI-TOF

**Age**	**Underlying condition**	**Sepsis source**	**Empirical therapy**	**DIMT result**	**Changed prompted by DIMT**
5 years	Vesicoureteral reflux and recurrent urinary tract infection	Urosepsis	Ceftriaxone	*E. faecalis*	Narrowed to ampicillin
5 months	Coffin-Siris syndrome, intubated & ventilated, long PICU stay	VAP	Vancomycin	*P. aeruginosa*	Meropenem added
12 months	Tufting enteropathy, PICC in place, TPN-dependent	CLABSI	Vancomycin	*E. cloacae*	Meropenem added
7 weeks	Lung malformation, enteroviral myocarditis, intubated & ventilated, long NICU stay	VAP	Vancomycin + pip-taz	*S. marcescens*	Escalated to meropenem
13 years	Stuve-Wiedemann syndrome, bone dysplasia, severe scoliosis	HAP	Pip-taz	non- typeable *H. influenzae*	Narrowed to ceftriaxone

3 weeks	Anorectal malformation, colostomy	Surgical wound infection	Meropenem	*E. faecalis*	Ampicillin added
16 years	Duchenne muscular PICC & tracheostomy in place, non-invasive ventilation in PICU	SWAS	Pip-taz	*E. cloacae*	Escalated to meropenem
8 years	Short bowel syndrome, port-a-cath in place, TPN-dependent	CLABSI	Vancomycin + ceftriaxone	*E. cloacae*	Escalated to meropenem
7 weeks	Prematurity, NEC, laparotomy, Ileostomy in place, long NICU stay	VLOGBSS	Vancomycin + pip-taz	*S. agalactiae*	Narrowed to penicillin + gentamicin
5 months	Short bowel syndrome, PICC & ileostomy in place, TPN-dependent	CLABSI	Vancomycin	*E. aerogenes*	Meropenem added
6 weeks	Pyloric atresia , laparotomy, PICC in place, long NICU stay	CLABSI	Cloxacillin + gentamicin	*S. capitis*	Escalated to vancomycin
4 weeks	CHD, cardiac catheterization, CVC in place, intubated & ventilated, long PICU stay	SWAS	Teicoplanin + gentamicn	*E. faecalis*	Narrowed to ampicillin + gentamicin

6 years	Previously healthy	Acute Osteomyelitis	Ceftriaxone	*S. pyogenes*	Narrowed to penicillin + clindamycin
11 years	Acute lymphoblastic leukemia, port-a-cath in place, neutropenia, ongoing MSSA sepsis	CLABSI	Cefazolin + amikacin	*E. cloacae*	Meropenem added
3 weeks	Hirschsprung's disease Intestinal resection, Ileostomy, PICC in place, TPN	SWAS	Vancomycin + meropenem	*E. faecalis*	Narrowed to ampicillin + gentamicin
5 months	Omenn's syndrome, recurrent enterococcal sepsis, sepsis PICC in place, TPN	CLABSI	Ampicillin + gentamicin	*P. aeruginosa*	Escalated to pip-taz
3 months	Hirschsprung's disease Intestinal resection, Ileostomy, PICC in place, TPN	SWAS	Vancomycin + gentamicin	*E. faecalis*	Narrowed to ampicillin + gentamicin
5 months	Multiple neonatal co-morbidities, bronchiolitis, epilepsy, PICC in place, long PICU stay	SWAS	Off antibiotics	*P. aeruginosa*	Started on pip-taz

16 months	Congenital muscular dystrophy, viral exacerbation CLD<br>PEG feeding tube in place	SWAS	Ceftriaxone	*E. faecalis*	Narrowed to ampicillin
1 month	Ileal atresia, necrotic bowel, laparotomy, adhesiolysis	Peritonitis	Vancomycin + gentamicin + metronidazole	*E. faecalis*	Narrowed to ampicillin + gentamicin + metronidazole
2 months	Ileal atresia, laparotomy, PICC in place, TPN	CLABSI	Ampicillin + gentamicin	*K. pneumoniae*	Escalated to meropenem
11 months	Acute myeloid leukemia, induction chemotherapy, port-a-cath, non-neutropenic fever	CLABSI	Ceftriaxone	*E. faecium*	Escalated to teicoplanin
2 years	Short bowel syndrome, PICC in place, TPN	CLABSI	Off antibiotics	*E. faecalis*	Started on ampicillin + gentamicin
16 months	Previously healthy	Infected burn wound	Off antibiotics	*S. aureus*	Started on cloxacillin
23 months	Brain tumor, port-a-cath, non-neutropenic sepsis	CLABSI	Vancomycin + Ceftriaxone	*S. marcescens*	Escalated to meropenem

3 months	IUGR	VLOGBSS	Off antibiotics	*S. agalactiae*	Started on ampicillin
10 months	Previously healthy	OB	Off antibiotics	*S. pneumoniae*	Started on ceftriaxone

DIMT: Direct identification by MALDI-TOF. ICU: Pediatric Intensive Care Unit; VAP: Ventilator-associated pneumonia; PICC: Peripherally inserted central catheter; TPN: Total parenteral nutrition; CLABSI: Central line associated blood stream infection; NICU: Neonatal Intensive Care Unit; HAP: Hospital-acquired pneumonia; Pip-taz: Piperacillin-tazobactam; SWAS: Sepsis without an apparent source; NEC: Necrotizing enterocolitis; VLOGBSS: Very late-onset group B *Streptococcus* sepsis; CHD: Congenital heart disease; CVC: Central venous catheter; MSSA: Methicillin-susceptible *Staphylococcus aureus*; CLD: Chronic lung disease; PEG: percutaneous endoscopic gastrostomy; IUGR: Intrauterine growth restriction.

Supplementary Table 5. Missed opportunities to optimize treatment in blood stream infections based on direct identification by MALDI-TOF

Age	Underlying condition	Sepsis source	Empirical therapy	DIMT result	Treatment after notification of DIMT
12 months	Autoimmune enteropathy, PICC in place, TPN-dependent	CLABSI	Cefepime	*E. faecalis*	Switched to teicoplanin
12 days	31 weeker , gastric perforation, laparotomy	Peritonitis	Teicoplanin + meropenem	*C. albicans*	Amphotericin B added
5 years	Ohtahara syndrome, recurrent LRTI, recent meropenem course, PICC in place, ARF, hemodialysis	CLABSI	Vancomycin + cefepime	*E. faecalis*	Unchanged
5 weeks	Cystic fibrosis, meconium ileus & pseudocyst, laparotomy, ileostomy	Peritonitis	Vancomycin + pip-taz	*C. albicans*	Amphotericin B added
3 years	ACS, hydrocephalus, spina bifida, neurogenic bladder, ongoing ESBL bacteremia	Urosepsis	Ertapenem	*C. albicans*	Caspofungin added

17 months	Boring Opitz syndrome, Upper GI surgery, broad-spectrum antibiotic treatment, PEG feeding tube in place	Abdominal collection	Meropenem	*C. albicans*	Amphotericin B added
4 weeks	28 weeker, CLD, NEC, laparotomy, ileostomy	Abdominal collection	Pip-taz	*S. marcescens*	Unchanged
4 weeks	31 weeker, anorectal malformation, abdominal surgery, colostomy	LONS	Cloxacillin + Gentamicin	*S. marcescens*	Unchanged
3 months	Congenital ichthyosis, primary immunodeficiency	SWAS	Vancomycin + meropenem	*E. faecalis*	Unchanged
2 years	Previously healthy	Lymphadenitis	Clindamycin	*S. pyogenes*	Unchanged
4 months	SCID, pressure ulcer, ongoing *P. aeruginosa* bacteremia	SWAS	Ceftazidime-avibactam	*E. faecalis*	Vancomycin added
4 months	Short bowel syndrome, PICC in place, TPN-dependent, long NICU stay	SWAS	Cloxacillin + gentamicin	*E. faecalis*	Switched to vancomycin + gentamicin

4 months	Prematurity, cholestatic jaundice, laparotomy, stoma closure, PICC in place, long NICU stay	SWAS	Vancomycin + pip-taz	*E. cloacae*	Unchanged

DIMT: Direct identification by MALDI-TOF. PICC: Peripherally inserted central catheter; TPN: Total parenteral nutrition; CLABSI: Central line associated blood stream infection; LRTI: Lower respiratory tract infection; ARF: Acute renal failure; Pip-taz: Piperacillin-tazobactam; ESBL: Extended spectrum beta-lactamase; PEG: percutaneous endoscopic gastrostomy; CLD: Chronic lung disease; NEC: Necrotizing enterocolitis; LONS: Late-onset neonatal sepsis; SWAS: Sepsis without an apparent source; SCID: Severe combined immunodeficiency; PICC: Peripherally inserted central catheter; TPN: Total parenteral nutrition; NICU: Neonatal Intensive Care Unit.
